# Serum Cytokine Profiling in Pancreatic Ductal Adenocarcinoma: A Pilot Study of Candidate Discriminators, Sex Differences and Altered Co-Regulation

**DOI:** 10.3390/biom16101492

**Published:** 2026-10-14

**Authors:** Jesús García-Gómez, Samuel Agredano-Pérez, Diana Alejandra Rubio-Delgado, Adriana Aguilar-Lemarroy, Luis Felipe Jave-Suárez, Sarah Naomi Guevara-Hernández, Alejandro Bravo-Cuéllar, Aníbal Samael Méndez-Clemente, Georgina Hernández-Flores, María Martha Villaseñor-García

**Affiliations:** 1 División de Inmunología, Centro de Investigación Biomédica de Occidente (CIBO), Instituto Mexicano del Seguro Social (IMSS), Guadalajara 44340, Jalisco, Mexico; jesus.garcia9891@alumnos.udg.mx (J.G.-G.); luis.jave@academicos.udg.mx (L.F.J.-S.); sarah.guevara6143@alumnos.udg.mx (S.N.G.-H.); abravocster@gmail.com (A.B.-C.); asamaelmendezc@gmail.com (A.S.M.-C.); 2 Programa de Doctorado en Ciencias Biomédicas, Centro Universitario de Ciencias de la Salud, Universidad de Guadalajara, Guadalajara 44340, Jalisco, Mexico; 3 Clínica de Tumores Gastrointestinales, Servicio de Oncología, Unidad Médica de Alta Especialidad, Centro Médico Nacional de Occidente, Instituto Mexicano del Seguro Social (IMSS), Guadalajara 44340, Jalisco, Mexico; samuel_agredano@hotmail.com; 4 Hospital Regional de Alta Especialidad, Instituto de Seguridad y Servicios Sociales de los Trabajadores del Estado (IS-SSTE), Tlajomulco de Zúñiga 45640, Jalisco, Mexico; arubio@oncologia.org.mx; 5 Unidad de Investigación Epidemiológica y en Servicios de Salud, Centro Médico Nacional de Occidente, Instituto Mexicano del Seguro Social (IMSS), Guadalajara 44340, Jalisco, Mexico; adriana.aguilar@academicos.udg.mx or adriana.aguilar@imss.gob.mx; 6 Programa de Doctorado en Microbiología Médica, Centro Universitario de Ciencias de la Salud, Universidad de Guadalajara, Guadalajara 44340, Jalisco, Mexico

**Keywords:** pancreatic ductal adenocarcinoma, cytokines, sex differences, candidate markers, IL-7, multiplex immunoassay

## Abstract

Pancreatic ductal adenocarcinoma (PDAC) is rarely detected before the disease is advanced, and existing serum markers are insufficient for early detection. Its systemic immune alterations are incompletely characterized and rarely examined by sex. We measured 19 serum cytokines by bead-based multiplex assay in 22 patients with PDAC and 16 controls, comparing groups overall and separately in women and men, and examined co-regulation among them. In the full cohort, interleukin-7 (IL-7) was the only cytokine to discriminate patients from controls after correction for multiple testing. Adjustment for age strengthened this association, and IL-7 matched the discrimination of carbohydrate antigen 19-9 (CA19-9). After stratification by sex, IL-7 remained the only cytokine higher in women, though it did not survive correction within that subgroup, whereas in men, eight innate, T-helper 17 (Th17) and type I interferon cytokines (IL-1α, IL-1RA, IL-31, IFN-α, IL-17A, IL-17F, TNF-α, and TNF-β) separated patients from controls, although the male control group was small. The correlation network present in controls was largely absent in patients, which instead showed few associations centered on IL-2, IL-5 and IL-6. These findings identify IL-7 as a candidate discriminator of PDAC and show that the cytokines separating patients from controls differed by sex, warranting validation in a larger, sex-balanced cohort that also measures sex hormones.

## 1. Introduction

Pancreatic ductal adenocarcinoma (PDAC) accounts for more than 90% of pancreatic cancers and ranks among the most lethal malignancies [1,2]. Early disease is usually asymptomatic: more than 80% of patients therefore reach diagnosis with locally advanced or metastatic disease that cannot be resected; together with the tumor’s aggressiveness and resistance to therapy, this holds the 5-year survival rate near 10% [1,3]. By 2040, it is projected to become the second most common cause of cancer death in the United States [4]. In Mexico, it ranks sixth among causes of cancer death [5], with a national mortality of about 4.3 per 100,000 [6]; in an eleven-year tertiary-center series from the region, most patients were diagnosed at stage III or IV, leaving only about a quarter with localized tumors amenable to curative surgery [7].

PDAC’s immune disturbance is confined neither to the tumor nor to a single pathway. Dynamic cross-talk between mutated cells and activated pancreatic stellate cells establishes a desmoplastic microenvironment fueled by ongoing local cytokine and growth factor signaling [8]. In the adaptive compartment, the T-helper 1 (Th1) arm that would oppose the tumor is displaced by a T-helper 2 (Th2) skew associated with shorter survival [9], while an IL-17-driven T-helper 17 (Th17) program is engaged already in preinvasive lesions [10]; regulatory signaling through IL-10 and regulatory T cells (Tregs) enforces immune escape [11]. Running alongside these is the homeostatic γc axis, whose principal mediator, IL-7, sustains T-cell development and the peripheral T-cell pool [12,13], an axis that has received far less attention in PDAC than the effector arms above. These changes reach circulation, where serum cytokine and biomarker profiles can help to characterize the disease and differentiate clinical stages [14,15], though evidence rests on few studies with heterogeneous panels.

Sex bears on PDAC at more than one level. Its incidence and mortality differ by sex [16], partly through modifiable exposures, tobacco and alcohol use and the metabolic burden of obesity and diabetes, that are established risk factors of the disease [1]. Sex also acts on immunity directly. Innate and adaptive responses vary between sexes, including in cytokine production, under the influence of sex hormones and X-linked genes [17,18], and in PDAC the systemic and local immune response is no exception [19]. A circulating cytokine profile intended as a biomarker therefore cannot be assumed to behave alike in men and women.

Currently, clinical monitoring of this disease relies on Carbohydrate Antigen 19-9 (CA19-9), the single circulating marker in routine clinical use and one established for following progression rather than for screening [20], but its elevation is not specific to cancer. In asymptomatic screening, fewer than 1% of raised CA19-9 values reflect PDAC [21]; elevations occur across a wide range of benign conditions, hepatic, gynecologic, pulmonary and endocrine [22], and in other gastrointestinal cancers [21]. At the other end, the 5–10% of people who are Lewis-antigen negative produce little CA19-9 and are missed regardless of tumor burden [21]. These shortfalls have driven a search for better circulating markers, from the addition of thrombospondin-2 to CA19-9 [23] and multi-marker serum signatures [24] to liquid biopsies pairing circulating tumor DNA with proteins [25] or profiling tumor-derived extracellular vesicles, by their protein content [26] or their RNA cargo [27,28]; early detection nonetheless remains unresolved. In PDAC, our group has found proliferation-linked polyamines and their rate-limiting enzyme ornithine decarboxylase raised in patients [29]. Cytokines extend that search with a signal grounded in the immune processes that drive the disease, an approach with which we have previously derived diagnostic candidates for ovarian lesions [30].

To identify circulating cytokines that distinguish PDAC from controls, we measured a panel spanning the innate, Th1, Th2, Th17, regulatory and homeostatic axes in serum, comparing the groups overall and separately in women and men, mapping their co-regulation and setting the resulting candidates against CA19-9. This is a discovery effort, meant to surface candidates and any sex-specific pattern among them, for validation in larger cohorts.

## 2. Materials and Methods

### 2.1. Study Participants

This case–control design is as follows: 22 patients with histopathologically confirmed PDAC were recruited at diagnosis from the Oncology Service of the Unidad Medica de Alta Especialidad (High Specialty Medical Unit), Centro Medico Nacional de Occidente (Western National Medical Center), Instituto Mexicano del Seguro Social (Mexican Social Security Institute) in Guadalajara, Mexico, from November 2019 to January 2025, each before any antineoplastic treatment began. The control group comprised 16 apparently healthy individuals recruited among voluntary donors at the blood bank of the same institution, selected to approximate the age and sex distribution of the patient group. All had passed the routine health evaluation required for blood donation. Participants in either group were not eligible if they had an autoimmune or chronic inflammatory disease, an active infectious process, or had received immunosuppressive or immunomodulatory therapy; controls additionally had no history of malignancy. Age and sex were recorded for all participants. Comorbidities and risk factors, clinical presentation, and serum CEA and CA19-9 were documented in the 22 patients and in 11 of the 16 controls, the remaining five having consented to sample donation but not to the accompanying questionnaire and laboratory tests; because controls were enrolled as healthy, they were recorded as free of the clinical signs assessed in patients. Clinical stage according to the 8th edition of the AJCC staging system [31], resectability, diagnostic procedures, hematological parameters at diagnosis, and the treatment received were recorded for patients only. Approval was granted by the National Scientific Research Committee (F-CNIC-2019-135, 13 November 2019) and the Local Health Research Committee 1305 of the Western Biomedical Research Center (Centro de Investigación Biomédica de Occidente) (R-2025-1305-040, 5 February 2025), in keeping with the Declaration of Helsinki, and every participant gave written consent before enrollment.

### 2.2. Sample Collection

Peripheral blood (5 mL) was obtained by venipuncture from patients at the Oncology Service and from controls at the blood bank and collected in protease-free tubes without anticoagulant. Tubes were labeled, placed in closed containers with double packaging, and transported to the Division of Immunology of the Western Biomedical Research Center (CIBO). Serum was separated by centrifugation and stored at −80 °C until cytokine determination.

### 2.3. Cytokine Quantification

Nineteen cytokines were measured in serum using two complementary bead-based multiplex immunoassays, each read on a different platform, together covering innate, Th1, Th2, Th17, regulatory and homeostatic cytokines.

Seven of these, IFN-α, IL-1α, IL-1RA, IL-7, IL-15, IL-31, and TNF-β, were measured with the ProcartaPlex™ Human Cytokine Panel 1C (7-plex; EPX070-10010-901, Thermo Fisher Scientific, Waltham, MA, USA), following the manufacturer’s protocol. Serum was incubated with antibody-coupled magnetic beads for 2 h; captured cytokines were then labeled with biotinylated detection antibodies (60 min) and streptavidin–phycoerythrin (15 min), with washes between steps. Beads were read on a Bio-Plex instrument (Bio-Rad, Hercules, CA, USA), and concentrations were interpolated from analyte-specific standard curves in Bio-Plex Manager v5.0 (Bio-Rad).

The remaining twelve, IL-2, IL-4, IL-5, IL-6, IL-9, IL-10, IL-13, IL-17A, IL-17F, IL-22, IFN-γ, and TNF-α, were measured with the LEGENDplex™ Human Th Cytokine Panel (12-plex, w/VbP V02; 741028, BioLegend, San Diego, CA, USA). Capture beads were incubated with serum per the manufacturer’s instructions and read on a Cytek™ Aurora spectral cytometer (Cytek Biosciences, Fremont, CA, USA), which distinguishes bead populations by size and fluorescence; at least 300 events were collected per analyte. Concentrations were derived from a run-matched standard curve in the LEGENDplex™ Data Analysis Suite (v2025-05-01; BioLegend). All values are expressed as pg/mL. In some participants, limited serum volume precluded measurement of the full panel.

### 2.4. Statistical Analysis

All analyses used Python 3.13.3 (NumPy 2.3.4, pandas 2.2.3, SciPy 1.16.0, statsmodels 0.14.6, scikit-learn 1.6.1), with plots produced in matplotlib 3.10.3 and seaborn 0.13.2. Readings below an assay’s detection limit were set to half the lowest positive concentration measured for that analyte.

Controls and PDAC patients were compared with the two-sided Mann–Whitney U test, and effect size was given as rank-based η^2^ (Cohen’s thresholds: 0.01, small; 0.06, medium; 0.14, large). To account for the 19 cytokines tested, the Benjamini–Hochberg false discovery rate (FDR) procedure was applied to the *p*-values; both the raw *p*-value and the adjusted q-value are reported for each analyte, with significance set at *q* < 0.05. Findings with *p* < 0.05 that did not survive correction are reported as nominally significant and treated as exploratory. Given reported sex differences in PDAC and in cytokine responses, this comparison was run in the full cohort and, separately, in the female and male subgroups, correcting within each. As sample volume limited some measurements, each cytokine was analyzed on all available data, with per-group *n* reported for every analyte.

Each cytokine’s ability to discriminate between the two groups was receiver operating characteristic (ROC) analysis. Areas under the curve (AUC) were obtained by the trapezoidal rule, with 95% confidence intervals from 1000 bootstrap resamples, and the Youden index defined the optimal cut-off, at which sensitivity, specificity, and accuracy are reported. Associations among cytokines were examined by Spearman correlation, computed separately within patients and within controls, with coefficients and FDR-adjusted q-values obtained across all 171 analyte pairs within each group. Correlations are reported for the full cohort.

Clinical and demographic variables were compared between groups with the two-sided Mann–Whitney U test for continuous variables and Fisher’s exact test for categorical variables, each category being tested against all others combined; *p*-values were corrected by the Benjamini–Hochberg procedure across the 12 primary comparisons, one per variable, while the per-level breakdowns nested beneath a variable are descriptive and carry raw *p*-values only. The association between IL-7 and group was examined by logistic regression, with IL-7 entered as log_10_-transformed concentration and the odds ratio expressed per one standard deviation, in models fitted unadjusted, adjusted for age, and adjusted for age and sex; the relation between IL-7 and age was assessed by Spearman correlation within each group. Serum IL-7 was compared across clinical stage groups by the Kruskal–Wallis test and between patients with and without metastasis at diagnosis by the Mann–Whitney U test. CA19-9 and IL-7 were compared in the participants in whom both had been measured, each marker alone and the two together entered as log_10_-transformed predictors in unpenalized logistic models without an interaction term; discrimination is reported both on the participants used to fit each model and under leave-one-out cross-validation, and the models were compared by DeLong’s test, with differences in area expressed relative to CA19-9.

## 3. Results

### 3.1. Study Population and Cytokine Differences in the Overall Cohort

The characteristics of the 16 controls and 22 patients are summarized in Table 1. Patients were older (median 57.0 [53.2–61.0] versus 50.0 [46.2–53.2] years; *q* = 0.0042), while the sex distribution was comparable, and diabetes, hypertension, obesity, alcohol use, smoking status and family history of cancer did not differ; smoking of more than 16 cigarettes per day was confined to six patients. Jaundice was present in 16 patients (72.7%) and cachexia in 15 (68.2%). Both tumor markers were raised in PDAC, CEA at 4.2 [1.9–10.1] versus 1.1 [1.0–1.3] ng/mL (q = 0.0042) and CA19-9 at 173.0 [19.5–1160.8] versus 2.5 [2.5–7.0] U/mL (*q* = 0.0008). Most patients presented with advanced disease: 14 (63.6%) had stage IV tumors and metastasis at diagnosis, 16 (72.7%) were unresectable, and all received chemotherapy, most often FOLFIRINOX (13, 59.1%).

We then compared the serum cytokine profiles of the two groups. Of the 19 cytokines measured, only IL-7 was elevated in PDAC after correction, with IL-1α higher at nominal significance; the remaining seventeen were comparable between groups (Figure S1).

ROC analysis identified IL-7 as the stronger discriminator (AUC 0.831, 95% CI 0.680–0.959) over IL-1α (AUC 0.724, 95% CI 0.553–0.881), with Youden cut-offs of 49.6 and 11.2 pg/mL, respectively (Figure 1a). At these thresholds, IL-7 classified the groups with 77% sensitivity, 88% specificity, and 82% accuracy, against 64%, 81%, and 71% for IL-1α. IL-7 was higher in PDAC (median 65.5 vs. 30.7 pg/mL; *p* < 0.001, *q* = 0.011), a large effect (η^2^ = 0.321), with most patients above and most controls below the cut-off (Figure 1b). IL-1α rose more modestly (median 11.5 vs. 7.9 pg/mL; *p* = 0.019, η^2^ = 0.147) and did not survive FDR correction (*q* = 0.183; Figure 1c).

Since the patients were older than the controls (Table 1), the separation achieved by IL-7 was re-examined with adjustment (Figure 2). IL-7 did not track age within either group (controls ρ = 0.204, *p* = 0.447; patients ρ = −0.352, *p* = 0.108). Each standard deviation of log_10_(IL-7) multiplied the odds of PDAC by 3.25 (95% CI 1.19–8.87; *p* = 0.022) unadjusted, 3.87 (1.42–10.54; *p* = 0.008) after adjustment for age, and 4.21 (1.44–12.29; *p* = 0.008) after adjustment for age and sex.

Within the 22 patients, IL-7 was examined against disease extent (Figure 3). Median concentrations rose across stage groups, from 35.5 pg/mL in stages I–II (n = 3) to 58.7 pg/mL in stage III (n = 5) and 73.2 pg/mL in stage IV (n = 14), with a large effect size that did not reach significance (H = 4.30, *p* = 0.116, η^2^ = 0.204). Grouping the patients by metastasis at diagnosis, which sets the same 14 stage IV patients against the remaining 8, gave 73.2 against 49.5 pg/mL (U = 27.5, *p* = 0.056, η^2^ = 0.180). With three patients in the earliest stage group, these comparisons are underpowered and do not exclude an association between IL-7 and disease extent.

The discrimination achieved by IL-7 was then set against that of CA19-9 in the 33 participants carrying both markers, the 22 patients and the 11 controls with clinical data. Both markers, alone and in combination, were fitted as logistic models on log10-transformed concentrations (Figure 4). Evaluated on the participants used to fit them, CA19-9 reached an AUC of 0.901 (95% CI 0.787–0.984), IL-7 an AUC of 0.860 (0.694–0.975), computed on this reduced control set rather than on all 16 controls as in Figure 1, and the combination an AUC of 0.979 (0.931–1.000) (Figure 4a). Under leave-one-out cross-validation, the same three models gave 0.851 (0.703–0.970), 0.831 (0.662–0.958) and 0.917 (0.812–1.000), respectively (Figure 4b). Taking CA19-9 as the reference, IL-7 did not differ from it in either panel (ΔAUC −0.041, *p* = 0.648 apparent; −0.021, *p* = 0.853 cross-validated), and the combination scored above it without reaching significance (ΔAUC +0.079, *p* = 0.074 apparent; +0.066, *p* = 0.394 cross-validated).

### 3.2. Cytokine Differences in the Female Subgroup

Because both PDAC risk and cytokine responses differ between the sexes, we next analyzed women and men separately. Among the 22 women (11 controls, 11 PDAC), IL-7, the discriminator identified in the overall cohort, was the only cytokine nominally higher in female PDAC patients, with no difference across the remaining eighteen (Figure S2).

By ROC, IL-7 (Figure 5a) separated PDAC from controls with an AUC of 0.839 (95% CI 0.625–0.991) and a Youden cut-off of 54.2 pg/mL, giving 73% sensitivity, 91% specificity, and 82% accuracy. Concentrations were higher in the PDAC group (median 67.7 vs. 30.7 pg/mL; *p* = 0.007), a large effect (η^2^ = 0.345); the difference reached nominal significance but did not survive FDR correction (*q* = 0.142; Figure 5b).

### 3.3. Cytokine Differences in the Male Subgroup

In contrast to the female subgroup, the male cohort (5 controls, 11 PDAC) revealed a much broader and distinct profile: eight cytokines were elevated in PDAC and remained significant after FDR correction, while IL-7, the discriminator in the overall and female cohorts, did not differ between groups (Figure S3).

Four markers separated the groups almost completely (Figure 6). IL-31 (median 662.6 vs. 170.4 pg/mL), TNF-β (241.6 vs. 40.8 pg/mL), and IL-1RA (2387.2 vs. 1297.0 pg/mL) were each higher in PDAC with no overlap between groups, giving complete discrimination (AUC 1.000, 95% CI 1.000–1.000; all *q* = 0.012, η^2^ = 0.647; Figure 6a–c). IFN-α followed the same pattern with only minimal overlap (18.1 vs. 12.4 pg/mL; AUC 0.991, 95% CI 0.949–1.000; *q* = 0.012, η^2^ = 0.624; Figure 6d).

The remaining four were also elevated in male PDAC but separated the groups less sharply, in a graded series (Figure 7). IL-1α discriminated best of these (median 11.2 vs. 3.1 pg/mL; AUC 0.955, 95% CI 0.849–1.000; *q* = 0.016, η^2^ = 0.535; Figure 7a). The two Th17 cytokines followed closely and behaved alike: IL-17A (27.1 vs. 4.2 pg/mL; AUC 0.944, 95% CI 0.831–1.000; *q* = 0.022, η^2^ = 0.547; Figure 7b) and IL-17F (48.7 vs. 7.8 pg/mL; AUC 0.933, 95% CI 0.785–1.000; *q* = 0.026, η^2^ = 0.520; Figure 7c), each a large effect. TNF-α showed by far the widest gap in absolute concentration (2710.7 vs. 234.8 pg/mL) yet the lowest AUC of the four (0.911, 95% CI 0.700–1.000; *q* = 0.038, η^2^ = 0.468; Figure 7d), its broad spread across patients keeping the groups from fully separating. IL-17A, IL-17F and TNF-α were measured in 9 of the 11 male patients.

Across all eight cytokines, effect sizes were large (η^2^ 0.47–0.65) and discrimination was high (AUC 0.91–1.00), with IL-31, TNF-β, and IL-1RA separating the groups completely. Given only five male controls, the complete separations in particular should be read with caution.

### 3.4. Correlation Structure Among Cytokines

Beyond which cytokines differed between groups, we asked whether their co-regulation also differed. We computed pairwise Spearman correlations among the 19 cytokines separately within PDAC patients and within controls, judging significance on FDR-adjusted q-values across all 171 pairs (Figure 8); every significant correlation was positive.

Within PDAC, 10 of the 171 pairs reached significance (Figure 8a), forming two clusters and three isolated pairs. One linked TNF-α to IL-17A (ρ = 0.85, *q* < 0.001) and IL-17F (ρ = 0.73, q = 0.008), with the two IL-17 cytokines correlated (ρ = 0.70, *q* = 0.012). The other centered on IL-2, which correlated with IL-13 (ρ = 0.74, *q* = 0.008), IL-5 (ρ = 0.72, *q* = 0.012) and IL-6 (ρ = 0.65, *q* = 0.044), IL-5 and IL-6 being themselves strongly correlated (ρ = 0.94, *q* < 0.001). The isolated pairs were IL-10 with IL-9 (ρ = 0.76, *q* = 0.007), IL-1α with IFN-α (ρ = 0.70, *q* = 0.008), and TNF-β with IL-31 (ρ = 0.66, *q* = 0.016).

Within controls, the network was far denser, with 27 significant pairs (Figure 8b). One cluster centered on IL-1α, joining TNF-β, IL-31, IFN-α, and IL-1RA (TNF-β with IL-31, ρ = 0.90; TNF-β with IL-1α, ρ = 0.86; IL-31 with IFN-α, ρ = 0.81; TNF-β with IL-1RA, ρ = 0.77; all *q* ≤ 0.006), and extending to IL-4, IFN-γ and IL-17F. A second, broad cluster connected IFN-γ and IL-4 to the IL-17 cytokines and TNF-α (IFN-γ with IL-4, ρ = 0.92; IL-17F with IL-4, ρ = 0.92; TNF-α with IFN-γ, ρ = 0.90; IFN-γ with IL-17A, ρ = 0.88; all *q* < 0.001). Six of these pairs recurred in patients: the TNF-α–IL-17 triad, IL-13 with IL-2, IL-1α with IFN-α, and TNF-β with IL-31. The remaining twenty-one, including every IFN-γ and IL-1RA link, were absent, whereas the links among IL-2, IL-5 and IL-6 and the IL-10–IL-9 pair had no counterpart in controls. IL-7 was not significantly correlated with any other cytokine in either group.

## 4. Discussion

Pancreatic ductal adenocarcinoma is closely associated with inflammation and its inflammatory signaling cooperates with oncogenic KRAS to initiate pancreatic neoplasia [32]. These immune alterations are not confined to the tumor; multiplex serum cytokine measurements can discriminate patients with PDAC from controls [14]. We therefore profiled circulating cytokines across the innate, Th1, Th2, Th17, regulatory, and homeostatic axes, seeking markers that distinguish patients from controls. Sex differences are documented both in PDAC, including its systemic immune response [19], and in immune regulation more broadly [18]; we therefore assessed whether these alterations differed between sexes.

IL-7 was the only cytokine to separate PDAC from controls after correction, and it showed the highest discriminative performance of any marker in the full cohort. IL-7 is a γc-family cytokine that, under physiological conditions, acts through the IL-7 receptor to drive the development of T cells and to maintain their peripheral pool [12,13]. Its receptor has already been proposed as a diagnostic marker in this disease: IL-7R transcripts are raised in the peripheral blood mononuclear cells of patients with PDAC [33], and in a validation cohort of 522 individuals, IL-7R separated PDAC not only from healthy controls but also from benign pancreatic disease and from other malignancies, with further improvement when combined with CA19-9 [34]. The ligand has been examined far less. In colorectal cancer, patients carry higher circulating IL-7 than controls, and it rises further with lymph-node involvement and distant metastasis [35], and in PDAC circulating IL-7 is prognostically relevant, with higher post-chemotherapy concentrations independently associated with shorter overall survival [36], but its diagnostic value has not been established. Our results add that dimension to these observations, identifying circulating IL-7 as the most robust discriminator of PDAC from controls in this cohort, as has been reported for its receptor.

In our cohort, the patients were older than the controls, but age did not account for this signal; IL-7 did not track age within either group, and adjusting for age, and for age and sex, increased the odds associated with it, although at this sample size those adjusted models serve only as a sensitivity check. Within the patients, IL-7 rose across stage groups without reaching significance, so the cohort neither establishes nor excludes a relation to disease extent; the same question has been answered negatively for the receptor, which did not differ between resectable and unresectable PDAC [34], while in colorectal cancer circulating IL-7 does rise with nodal and distant spread [35]. Against CA19-9, IL-7 performed comparably, and the two together scored higher than CA19-9 alone without the difference reaching significance, consistent with the gain reported when IL-7R is combined with CA19-9 [34]. That comparison rests on 33 participants and is not the basis for a claim of added value. The IL-7 signal was present in the overall cohort and, at nominal significance, in women but absent in men, the first sign of a broader divergence between the sexes.

In the male subgroup, IL-1α and IL-1RA were both elevated in PDAC, with IL-1RA among the markers that separated the groups completely. IL-1α is over-expressed in human PDAC and, in cell-line and mouse models, acts as a tumor-cell-derived driver of the desmoplastic, inflammatory stroma through cancer-associated fibroblast activation, a mechanism linked to chemoresistance [37]; the elevated IL-1α we observed in male PDAC is consistent with engagement of this tumor-derived inflammatory program. The role of IL-1RA, the endogenous antagonist of IL-1, appears less straightforward. In a recent study, serum IL-1RA exceeded that of healthy donors in patients with PDAC, and higher IL1RN expression tracked shorter survival and more advanced disease, while accompanying mouse and in vitro experiments indicated that tumor-derived IL-1RA can suppress the activation of CD8+ T cells and their antigen-specific production of IFN-γ [38]. In contrast, human genetic analysis has linked constitutively higher IL-1RA to lower pancreatic cancer risk [39]. The raised circulating IL-1RA we observed in male PDAC is most consistent with the tumor-associated form seen in established disease rather than this protective, constitutive signal, though the supporting human evidence remains limited and the immunosuppressive mechanism confined to experimental models.

Elevated IL-17A in male patients implicates the Th17 axis, which has a pro-tumorigenic role in pancreatic cancer. An earlier serum study found IL-17A raised in patients with PDAC relative to healthy donors and raised further in advanced relative to early-stage disease [40]. In mouse models, IL-17A is needed for pancreatic preinvasive lesions to arise and to advance, and IL-17-producing cells are enriched in human precursor lesions [10]. In patients, infiltration by IL-17A-producing CD8+ T cells accompanies advanced stage and shorter survival, and IL-17A, acting together with TNF, induces the inflammatory cancer-associated fibroblasts that promote tumor growth [41]. The elevated circulating IL-17A we observed in male PDAC is consistent with systemic activity of this pro-tumorigenic axis.

The elevation of IL-17F was likewise confined to male patients. Unlike IL-17A, it shows no consistent direction in cancer: it is tumor-suppressive in some malignancies, including hepatocellular carcinoma [42] and oral tongue squamous cell carcinoma [43], yet elevated in prostate cancer [44] and tumor-promoting in bladder cancer [45]; within colorectal cancer, the reports directly conflict, describing IL-17F as both downregulated and protective [46] and upregulated and invasion-promoting [47]. In PDAC specifically, a larger multiplex serum study found IL-17F indistinguishable in patients and controls, and lower than in chronic pancreatitis [48], concordant with our overall analysis.

TNF-α was similarly raised in male patients and is among the most studied inflammatory mediators in pancreatic cancer. Tumor cells and pancreatic stellate cells both overexpress it, and it sustains a tumor-promoting inflammatory microenvironment, driving desmoplasia and chemoresistance, such that its neutralization reduces stromal density and improves chemotherapy response in preclinical models [49]. It also maintains the aggressive basal-like transcriptional state of PDAC via macrophage-driven lineage reprogramming, where elevated TNF-α accompanies shorter survival [50], and it cooperates with IL-17A to generate the inflammatory cancer-associated fibroblasts noted above [41]. Systemically, serum TNF-α has been reported above control concentrations in PDAC [51], and in a larger cohort it discriminated patients with moderate performance while carrying no prognostic value independent of established markers [15], although not all cohorts found a difference [48]. In our data, its elevation is consistent with this pro-tumorigenic axis.

Following the male-restricted pattern, TNF-β (lymphotoxin-α) was markedly elevated in male patients, though not in the overall cohort. Its role in cancer is scarcely examined, and in PDAC has received essentially no attention. In its secreted homotrimeric form, TNF-β engages TNFR1 and, in cell-based and biochemical assays, triggers apoptosis, necroptosis, and NF-κB/MAPK signaling with the same potency as TNF-α [52]. In one relevant study, TNF-β activated NF-κB in a colorectal cancer cell line and, like TNF-α, upregulated proliferative and invasive gene products (cyclin D1, MMP-9, CXCR4) while promoting growth and invasion [53]. Because secreted TNF-β signals through TNFR1 like TNF-α, its elevation could reflect the same inflammatory program, though this remains an inference.

IFN-α was also raised in men, and its role in PDAC is not clearly pro-tumorigenic. As a type I interferon, it can act against the tumor: directly, though weakly and only where the type I IFN receptor is well expressed, it inhibits the growth of PDAC cell lines [54], and when engaged through STING, it drives CXCR3-dependent antitumor T-cell immunity in mice [55]. Endogenous type I IFN is nonetheless normally suppressed in PDAC by oncogenic KRAS and MYC [56], while a tumor-intrinsic program, de-repressed through hypomethylation of endogenous retroelements, marks the aggressive basal-like subtype and independently predicts shorter survival in patients [57]. Circulating IFN-α has rarely been examined in PDAC, and we are not aware of a previous report of its elevation in patients. Against a background of habitual IFN suppression, it could reflect either a host antitumor response or the intrinsic IFN program of aggressive disease, and our data cannot distinguish between them.

IL-31 was found to be greatly increased in male patients relative to male controls. An IL-6-family cytokine released by Th2 lymphocytes and a range of other immune cells, including mast cells [58,59], IL-31 signals through the IL-31RA/OSMRβ complex and is best characterized as a mediator of pruritus and skin inflammation [58]. Its role in cancer is contradictory and tissue-dependent: in mouse models of colon and breast cancer, it suppresses tumor growth and angiogenesis [60], and it is reduced in gastric tumor tissue [61], whereas in endometrial cancer serum IL-31 is elevated and rises with tumor stage [62]. Even its effect on angiogenesis differs by setting, being antiangiogenic in those tumor models yet inducing the pro-angiogenic factor VEGF in airway epithelial cells [63]. IL-31 has not been studied in PDAC apart from a single serum survey which found it lower in patients than in controls [48]; the opposite of what we observe. Our elevation therefore conflicts with the only prior PDAC measurement, draws on thin literature and rests on the smallest control group, making it the least secure of the male-restricted signals.

A single feature links these male-restricted signals, which otherwise span unrelated pathways. Each was present at lower median concentrations in our male controls than in our female controls (Supplementary Figures S2 and S3), and against that quiet baseline a cancer-associated rise is more evident, separating patients from controls more readily than against a higher background. Much of that low baseline may be androgen-dependent. Testosterone suppresses Th17 activity [64], and the production of IFN-α by plasmacytoid dendritic cells [65], and circulating TNF-α rises in men when testosterone falls [66], consistent with what we observed in our male controls. Male pancreatic cancer may sharpen the contrast further, since hypogonadism and testosterone deficiency are common in these patients [67] and a fall in these hormones could reduce this suppression. The absence of these differences in women, who lack a comparable androgenic restraint to lift, is consistent with this account. Because this evidence comes from experimental and non-cancer settings and sex hormones were not measured here, the account remains a hypothesis, and the male signature is a lead to pursue rather than a difference we can claim.

Beyond their concentrations, the cytokines differed in how strongly they correlated with one another. Controls carried a connected network of positive correlations, denser than that of the patients. IFN-γ, which did not differ in concentration between the groups, anchored one of its clusters, correlated with IL-4, the IL-17 cytokines, and TNF-α, yet showed no significant correlation in patients. The loss, however, was uneven. A pro-inflammatory core persisted, TNF-α remaining correlated with IL-17A and IL-17F, the pairing that elsewhere drives inflammatory cancer-associated fibroblasts [41], now isolated from the broader Th1 network in which it was embedded in controls. The innate cluster of the control network, joining IL-1α, TNF-β, IL-31, IFN-α and IL-1RA, and comprising the markers of the male signature, was reduced to two pairs, TNF-β with IL-31 and IL-1α with IFN-α; every IL-1RA link was gone. Patients meanwhile carried associations that controls did not, among IL-2, IL-5 and IL-6 and between IL-10 and IL-9. A comparable weakening of serum cytokine correlations precedes glioma, where the tumor is thought to assume control of cytokine production from the host [68], and an early multianalyte study of PDAC likewise found circulating markers to be largely uncorrelated in patients [14]. With at most sixteen controls and twenty-two patients, these correlations remain exploratory.

## 5. Conclusions

In this study, serum cytokine profiling identified candidate markers of pancreatic cancer. IL-7 was the strongest single discriminator between patients and controls overall, and its association with disease was not explained by the age difference between the groups. A set of innate, Th17, and type I interferon cytokines separated the groups in men but not in the cohort as a whole; several of these are suppressed by androgens in health, and their elevation in male patients is consistent with a loss of that suppression. Beyond these differences in concentration, the coordination among cytokines that characterized controls was largely absent in patients, which carried a smaller and partly different set of associations.

The cohort is small, and power is correspondingly low, particularly in the male subgroup, where five controls make the complete separations unstable. The controls were also characterized less fully than the patients, with clinical variables documented in 11 of the 16. No acute-phase marker such as C-reactive protein was measured in either group, so the cytokine differences cannot be set against a general inflammatory response. Although CA19-9 was measured and compared, the cohort is underpowered for that comparison to be meaningful, and its cross-sectional design does not address prognosis. Because the markers that discriminated differed between the sexes, future work should be stratified by sex and include measurement of sex hormones to test the androgen hypothesis. Validation will require a larger, sex-balanced cohort in which these candidates are benchmarked against established biomarkers and patients are followed for survival and clinical outcome. Since the present design contrasts cancer with health alone, benign and premalignant pancreatic disease should also be included as comparators.

## Figures and Tables

**Figure 1:**
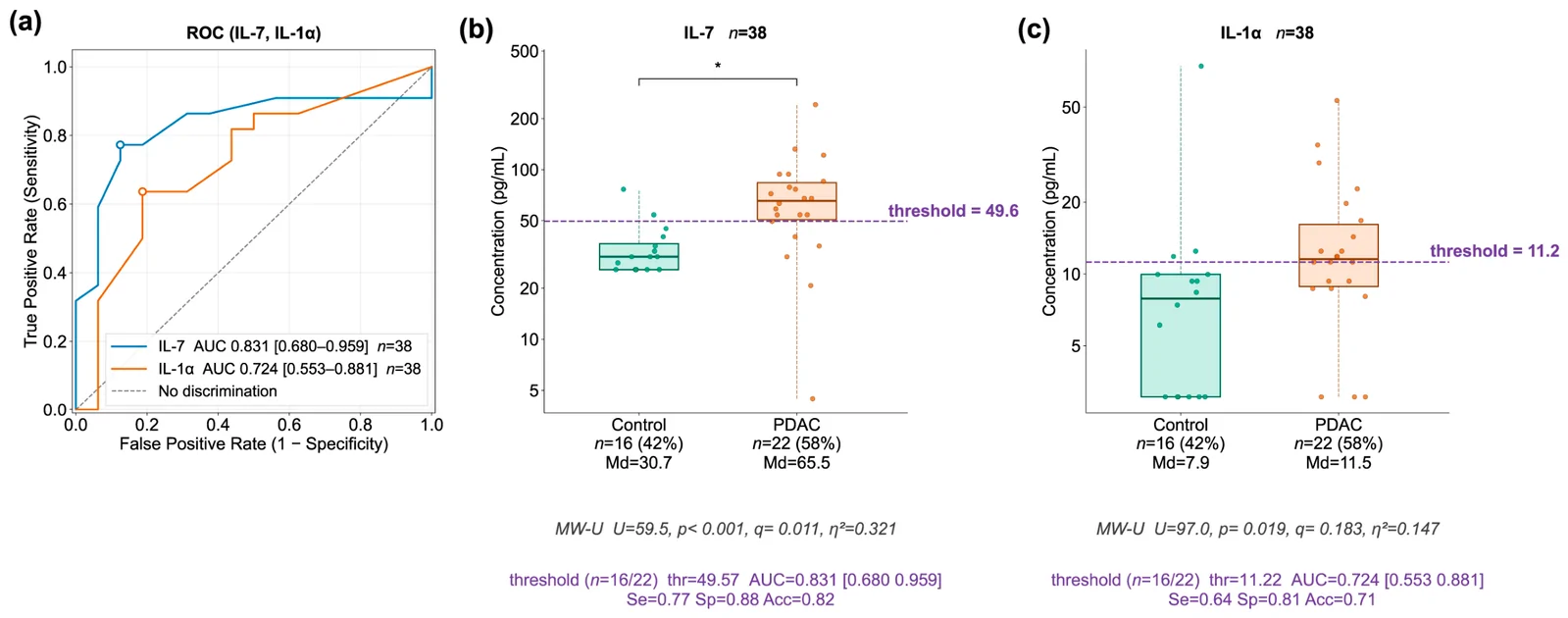
interleukin-7 and interleukin-1α (IL-1α) in the overall cohort (16 controls, 22 patients with pancreatic ductal adenocarcinoma, PDAC). (**a**) Receiver operating characteristic (ROC) curves for IL-7 and IL-1α; open circles mark the Youden cut-offs, with each area under the curve (AUC) and its bootstrap 95% confidence interval (CI) shown. Serum IL-7 (**b**) and IL-1α (**c**) by group on a log scale; boxes show the interquartile range and median, dashed vertical lines extend to the minimum and maximum, points are individual samples, and the horizontal dashed line marks the cut-off. Below each panel: per-group *n* and medians; the two-sided Mann–Whitney U test (U, raw *p*, and Benjamini–Hochberg *q*); the rank-based effect size η^2^; and the AUC (bootstrap 95% CI) with sensitivity, specificity, and accuracy at the cut-off. * *q* < 0.05.

**Figure 2:**
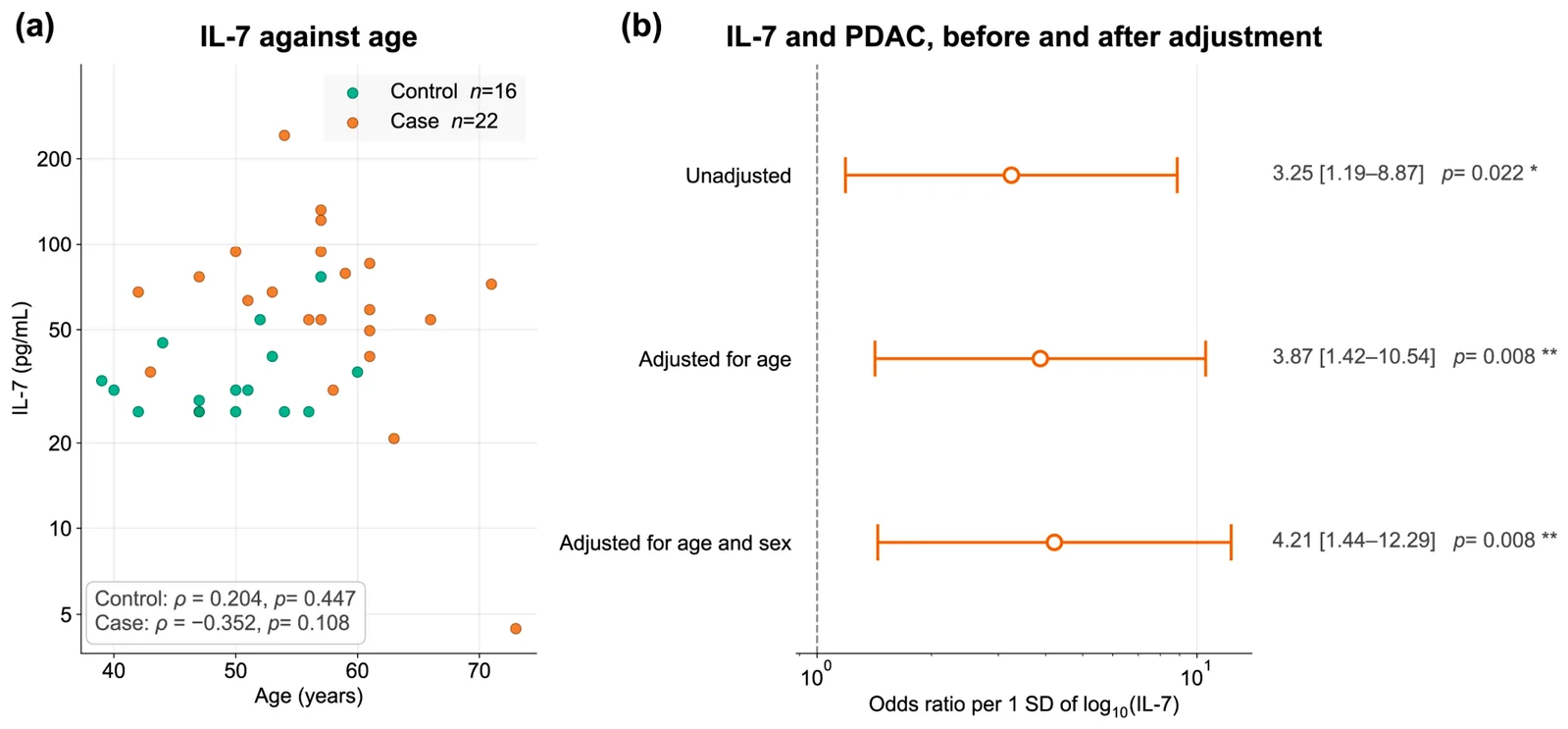
Serum IL-7 and age (16 controls, 22 PDAC). (**a**) IL-7, on a log scale, against age; each point is one participant, and the inset gives the within-group Spearman coefficient and its *p*-value. (**b**) Odds ratio for PDAC per one standard deviation of log_10_(IL-7), estimated by logistic regression unadjusted, adjusted for age, and adjusted for age and sex; open circles mark the point estimates, horizontal lines the 95% confidence intervals, and the dashed vertical line an odds ratio of 1. Each estimate, its interval and its *p*-value are printed to the right. * *p* < 0.05, ** *p* < 0.01.

**Figure 3:**
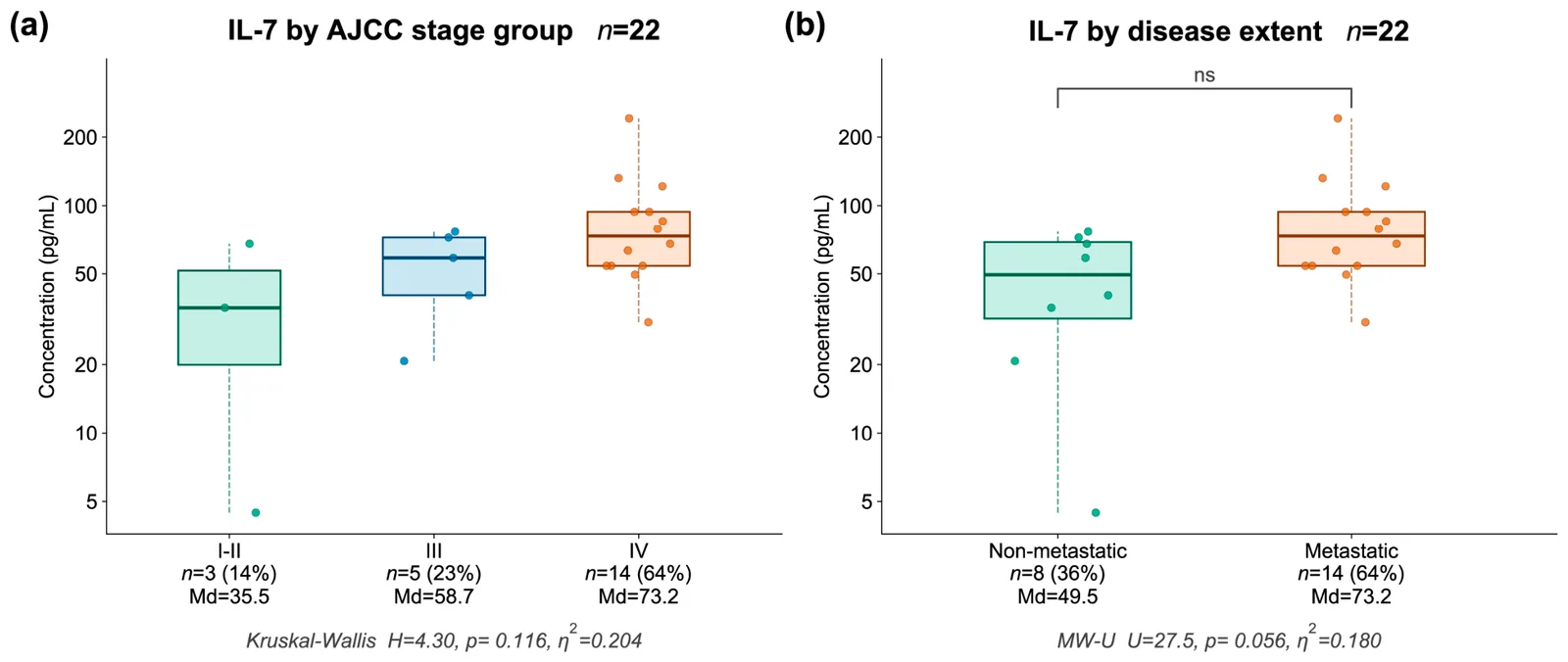
Serum IL-7 and disease extent in the 22 patients with PDAC. (**a**) IL-7 by American Joint Committee on Cancer (AJCC) stage group, with stages I and II pooled, and (**b**) IL-7 by the presence of metastasis at diagnosis; both on a log scale, with boxes showing the interquartile range and median, dashed vertical lines extending to the minimum and maximum, and points marking individual patients. Below each panel: the *n*, percentage and median of each group, followed by the test, the Kruskal–Wallis H in (**a**) and the two-sided Mann–Whitney U in (**b**), each with its raw *p* and the rank-based effect size η^2^. ns, not significant.

**Figure 4:**
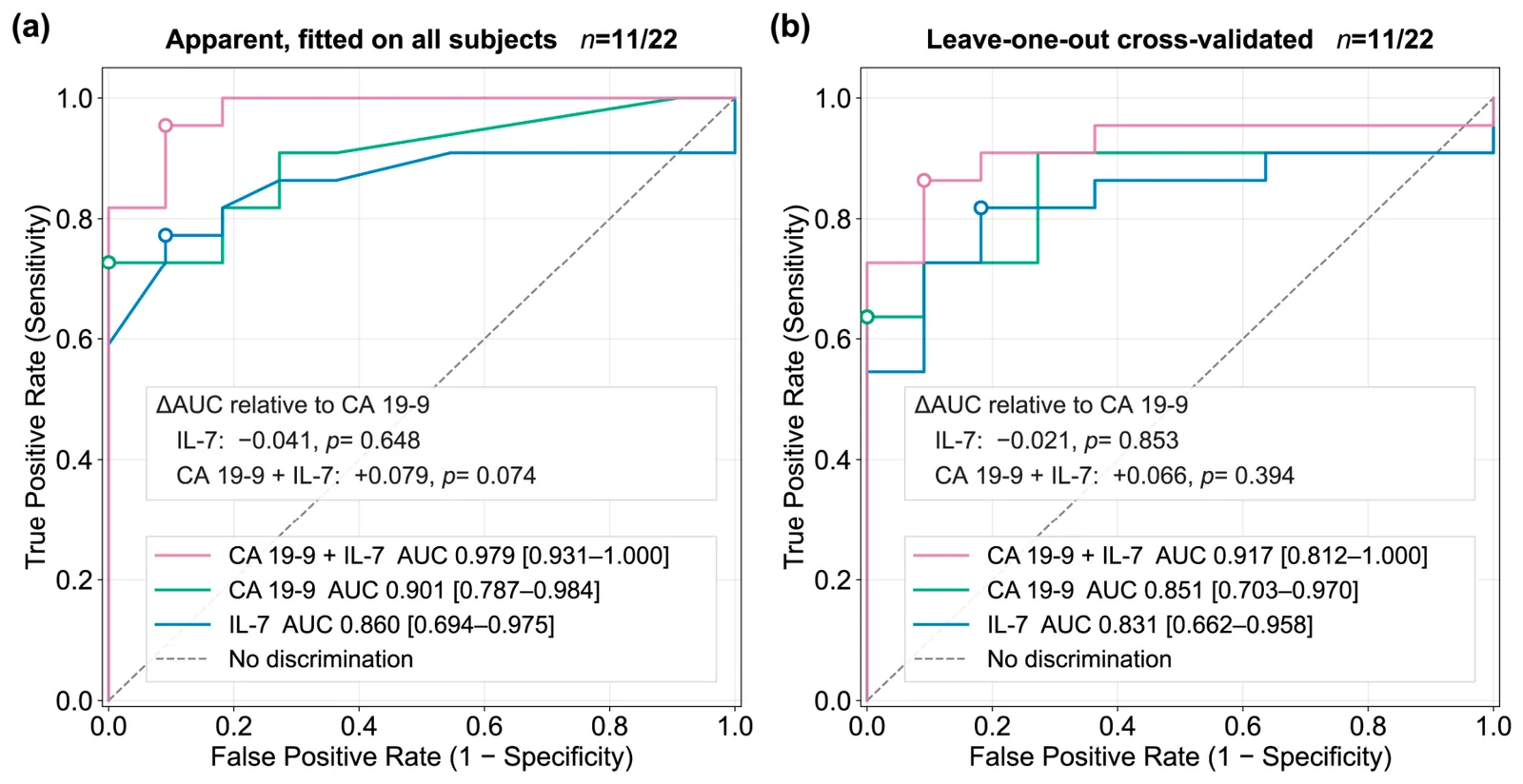
Discrimination by carbohydrate antigen 19-9 (CA19-9), IL-7 and the two combined, in the 33 participants in whom both markers were measured (22 PDAC, 11 controls). ROC curves for the three logistic models, each fitted on log10-transformed concentrations, showing (**a**) performance on the participants used to fit them and (**b**) leave-one-out cross-validated performance; for the single markers, the curve in (**a**) is identical to that of the untransformed marker. Open circles mark Youden cut-offs. Each legend gives the AUC with its bootstrap 95% confidence interval, and the diagonal marks no discrimination. The DeLong comparisons printed on each panel give the difference in area relative to CA19-9, so a positive value favors the model over CA19-9.

**Figure 5:**
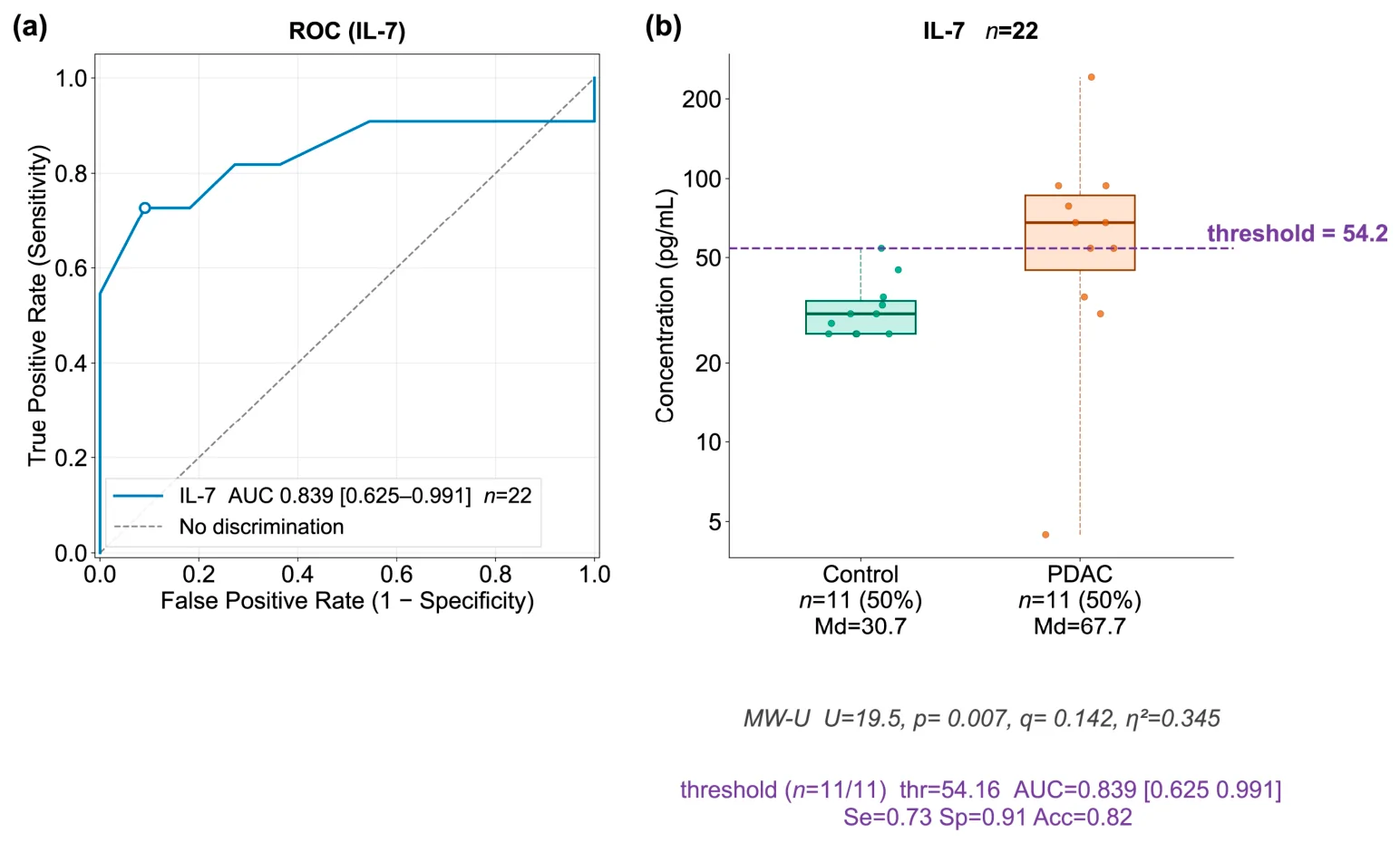
Circulating IL-7 in the female subgroup (11 controls, 11 PDAC). (**a**) ROC curve for IL-7; the open circle marks the Youden cut-off, with the AUC and its bootstrap 95% CI shown. (**b**) Serum IL-7 by group on a log scale; the box shows the interquartile range and median, dashed vertical lines extend to the minimum and maximum, points are individual samples, and the horizontal dashed line marks the cut-off. Below the panel: per-group *n* and medians; the two-sided Mann–Whitney U test (U, raw p, and Benjamini–Hochberg *q*); the rank-based effect size η^2^; and the AUC (bootstrap 95% CI) with sensitivity, specificity, and accuracy at the cut-off.

**Figure 6:**
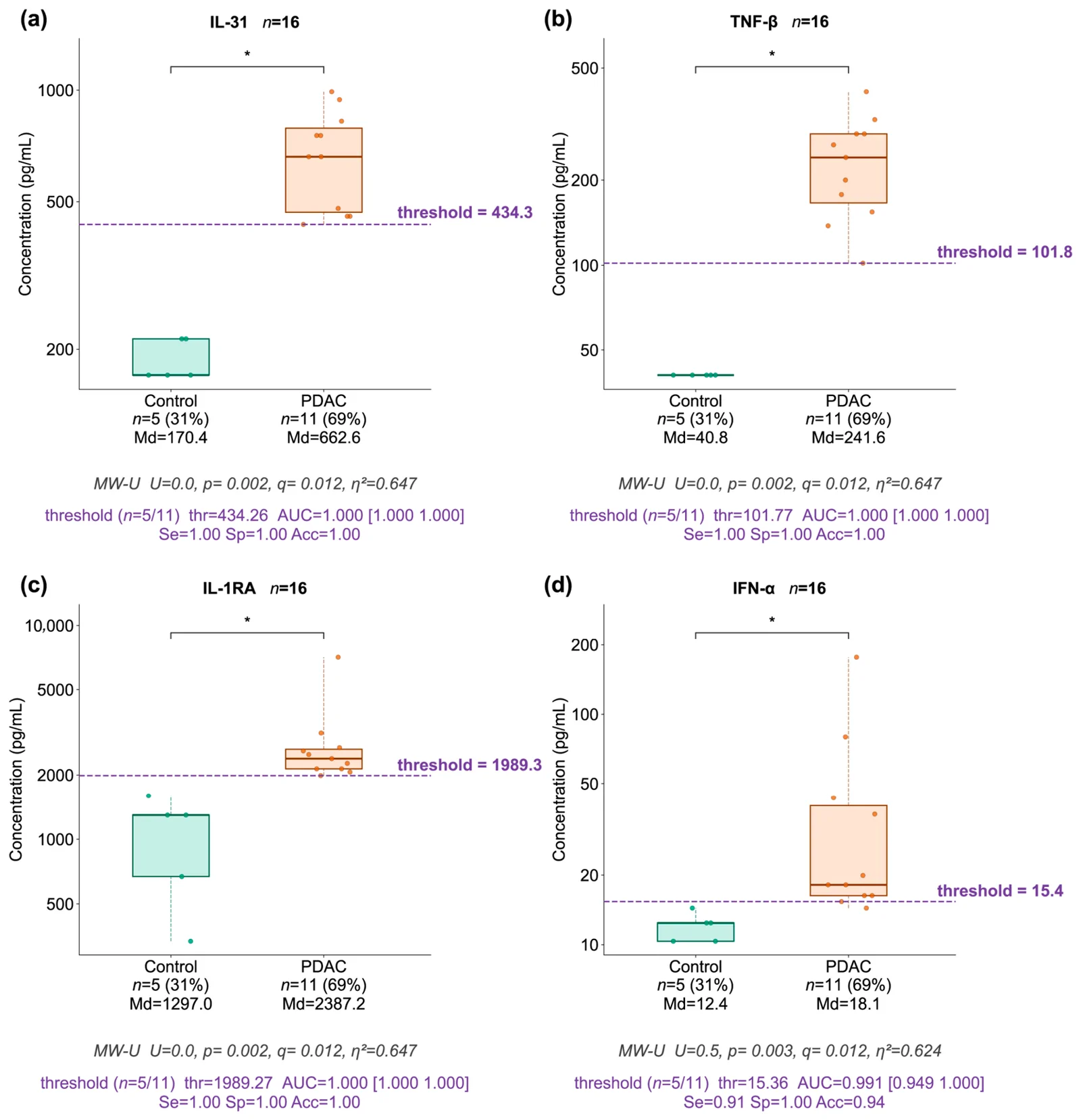
Innate and pro-inflammatory cytokines elevated in male PDAC. Serum IL-31 (**a**), TNF-β (**b**), IL-1RA (**c**), and IFN-α (**d**) by group on a log scale; boxes show the interquartile range and median, dashed vertical lines extend to the minimum and maximum, points are individual samples, and the horizontal dashed line marks the Youden cut-off. Below each panel: per-group *n* and medians; the two-sided Mann–Whitney U test (U, raw p, and Benjamini–Hochberg *q*); the rank-based effect size η^2^; and the AUC (bootstrap 95% CI) with sensitivity, specificity, and accuracy at the cut-off. * *q* < 0.05.

**Figure 7:**
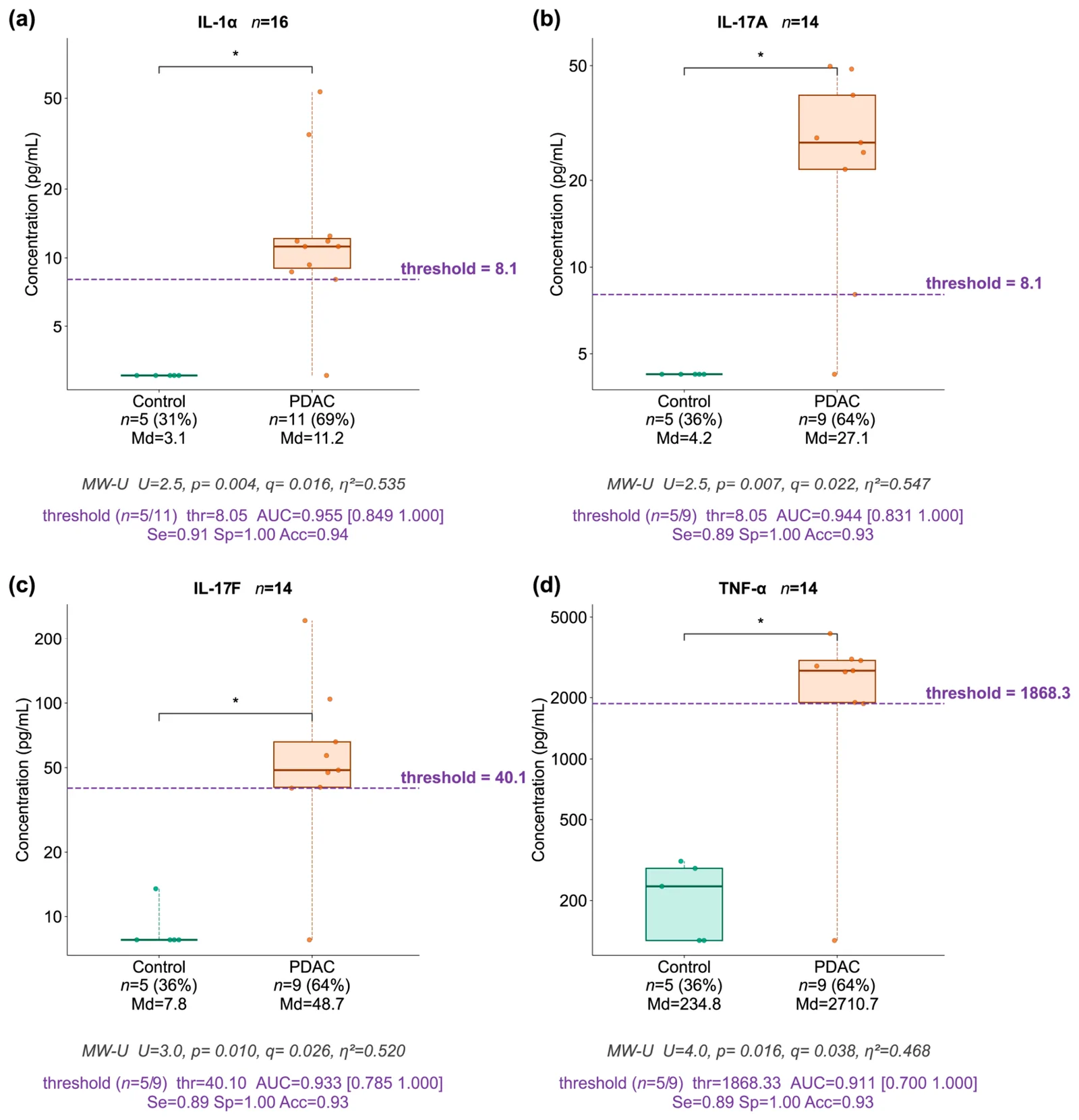
Additional cytokines elevated in male PDAC. Serum IL-1α (**a**), IL-17A (**b**), IL-17F (**c**), and TNF-α (**d**) by group on a log scale. Boxes, points, dashed cut-off line, and the statistics reported below each panel (per-group *n* and medians; the Mann–Whitney U test with U, raw *p*, and *q*; the rank-based effect size η^2^; and the AUC with 95% CI and sensitivity/specificity/accuracy) are as in Figure 6. * *q* < 0.05.

**Figure 8:**
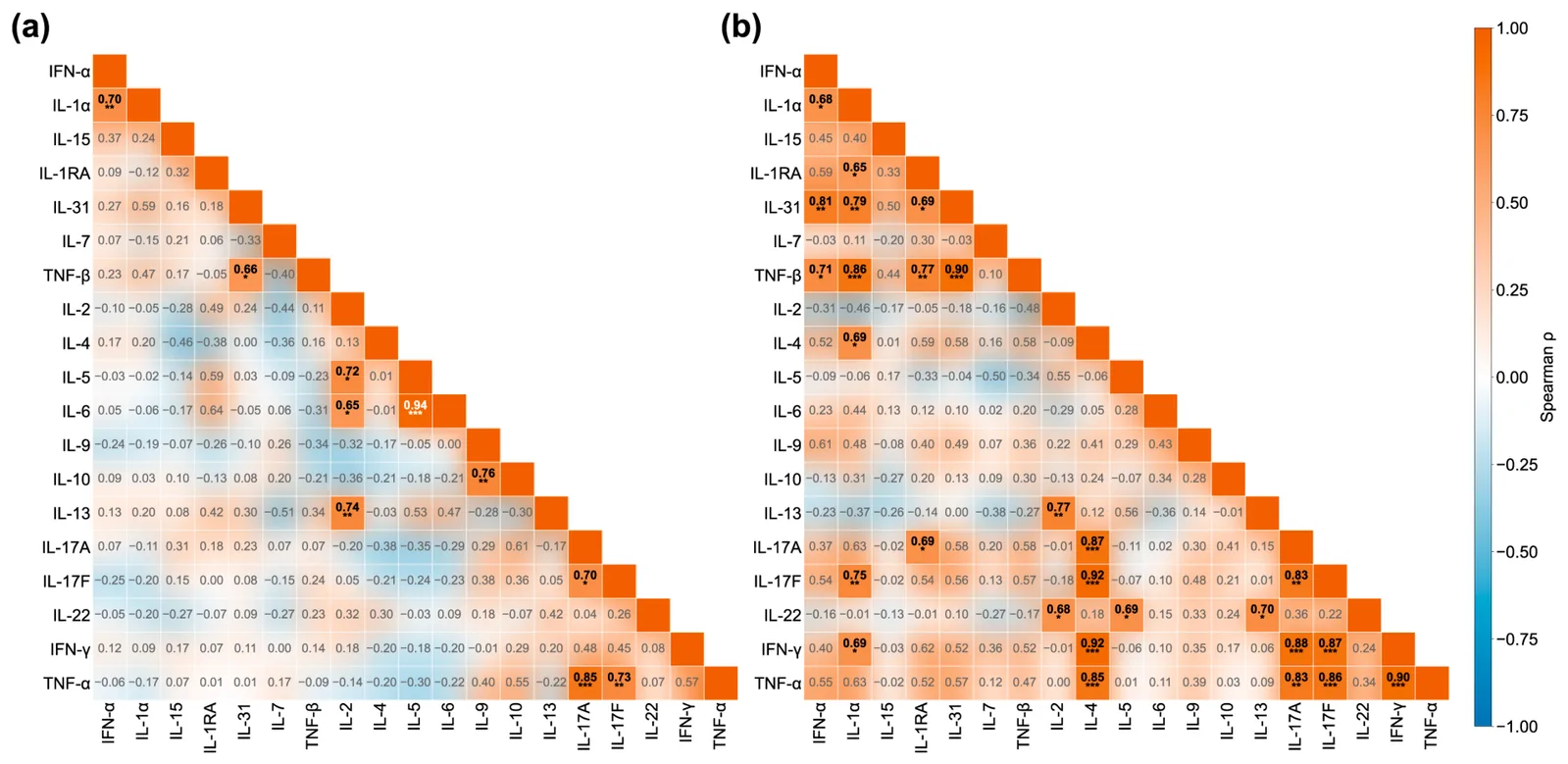
Cytokine co-regulation within groups. Spearman correlations among the 19 cytokines computed separately within PDAC patients (**a**) and controls (**b**). Each lower-triangle cell gives the coefficient ρ, colored from blue (negative) through white (zero) to orange (positive); the diagonal is self-correlation. Within each group, *p*-values were adjusted across all 171 pairs by the Benjamini–Hochberg procedure; non-significant cells (*q* ≥ 0.05) are blurred and their coefficients shown in grey, significant cells are marked (* *q* < 0.05, ** *q* < 0.01, *** *q* < 0.001).

**Table 1:** Demographic and clinical characteristics of the study population.

Variable	Control (*n* = 16)	PDAC (*n* = 22)	*p*-Value	*q*-Value
Demographics				
Age, years, median [IQR]	50.0 [46.2–53.2]	57.0 [53.2–61.0]	0.0018	0.0042
Age, years, mean +/− SD	49.3 +/− 6.1	57.2 +/− 7.8		
Age, years, range	39–60	42–73		
Sex, n (%)			0.3262	0.5593
Women	11 (68.8)	11 (50.0)		
Men	5 (31.2)	11 (50.0)		
Comorbidities and risk factors, n (%)				
Diabetes	2 (18.2)	8 (36.4)	0.4300	0.5733
Hypertension	1 (9.1)	6 (27.3)	0.3784	0.5677
Alcohol use	4 (36.4)	7 (31.8)	1.0000	1.0000
Family history of cancer	6 (54.5)	11 (50.0)	1.0000	1.0000
Smoking status			-	
Never smoked	8 (72.7)	14 (63.6)	0.7092	
Current smoker	3 (27.3)	7 (31.8)	1.0000	
Former smoker	0 (0.0)	1 (4.5)	1.0000	
Heavy smoking, >16 cigarettes/day	0 (0.0)	6 (27.3)	0.0770	0.1539
Body mass index category			-	
Normal weight	5 (45.5)	16 (72.7)	0.1490	
Overweight	4 (36.4)	1 (4.5)	0.0325	
Obesity class I	0 (0.0)	3 (13.6)	0.5343	
Obesity class II	2 (18.2)	1 (4.5)	0.2520	
Obesity class III	0 (0.0)	1 (4.5)	1.0000	
Obesity, class I-III	2 (18.2)	5 (22.7)	1.0000	1.0000
Clinical presentation, n (%)				
Jaundice	0 (0.0)	16 (72.7)	<0.0001	0.0008
Cachexia	0 (0.0)	15 (68.2)	0.0002	0.0008
Palpable mass	0 (0.0)	0 (0.0)	-	
No signs at presentation	11 (100.0)	5 (22.7)	<0.0001	
Tumor markers, median [IQR]				
CEA, ng/mL	1.1 [1.0–1.3]	4.2 [1.9–10.1]	0.0014	0.0042
CA19-9, U/mL	2.5 [2.5–7.0]	173.0 [19.5–1160.8]	0.0002	0.0008
Hematology at diagnosis, median [IQR]	Not assessed		-	
Hemoglobin, g/dL		12.3 [10.4–13.2]		
Leukocytes (WBC), ×10^9^/L		8.1 [6.5–9.0]		
Platelets, ×10^9^/L		295.5 [206.0–364.0]		
Tumor characteristics at diagnosis, n (%)	Not applicable		-	
Clinical stage (AJCC)				
Stage IB		2 (9.1)		
Stage IIB		1 (4.5)		
Stage III		5 (22.7)		
Stage IV		14 (63.6)		
Resectability				
Resectable		4 (18.2)		
Unresectable		16 (72.7)		
Borderline resectable		2 (9.1)		
Metastasis at diagnosis		14 (63.6)		
Biopsy performed		19 (86.4)		
ERCP				
Not performed		9 (40.9)		
Performed, conclusive		7 (31.8)		
Performed, non-conclusive		6 (27.3)		
Treatment, n (%)	Not applicable		-	
Surgery		3 (13.6)		
Radiotherapy		2 (9.1)		
Chemotherapy		22 (100.0)		
Chemotherapy regimen				
FOLFIRINOX		13 (59.1)		
Gemcitabine/cisplatin or gemcitabine/capecitabine		2 (9.1)		
Fluoropyrimidine/gemcitabine		7 (31.8)		
Chemotherapy intent				
Neoadjuvant		2 (9.1)		
Adjuvant		9 (40.9)		
Palliative		10 (45.5)		
Not specified		1 (4.5)		

Data are n (%) unless the row label states otherwise. Age and sex were available for all 16 controls, whereas the clinical variables were documented in 11 of them, and percentages are computed on the participants with data. Continuous variables were compared with the Mann–Whitney U test and categorical variables with Fisher’s exact test, both two-sided, each category being tested against all others combined. *p*-values are unadjusted; *q*-values are Benjamini–Hochberg false discovery rates across the 12 primary between-group comparisons, one per variable, while the per-level breakdowns nested beneath a variable are descriptive and carry a *p*-value only. Sections marked “Not assessed” were measurable in controls but were not recorded, and those marked “Not applicable” have no meaning in a healthy participant; neither admits a between-group comparison, which is what “-“ denotes in the *p*-value column. Controls were enrolled as healthy and are therefore recorded as free of clinical signs. AJCC, American Joint Committee on Cancer; CA19-9, carbohydrate antigen 19-9; CEA, carcinoembryonic antigen; ERCP, endoscopic retrograde cholangiopancreatography; IQR, interquartile range; PDAC, pancreatic ductal adenocarcinoma; SD, standard deviation; WBC, white blood cell count.

## Data Availability

The data presented in this study are not publicly available because they contain patient-level clinical information collected under the terms of the approving ethics committees. They are available from the corresponding authors upon reasonable request.

## Supplementary Materials

The following supporting information can be downloaded at https://www.mdpi.com/article/10.3390/biom16101492/s1. Figure S1: All serum cytokines in the overall cohort. Figure S2: All serum cytokines in the female subgroup. Figure S3: All serum cytokines in the male subgroup. Table S1: Raw serum cytokine values for all participants (pg/mL).

## Abbreviations

The following abbreviations are used in this manuscript:

AJCC	American Joint Committee on Cancer
AUC	Area Under the Curve
CA19-9	Carbohydrate Antigen 19-9
CD8	Cluster of Differentiation 8
CEA	Carcinoembryonic Antigen
CI	Confidence Interval
CIBO	Centro de Investigación Biomédica de Occidente
CXCR3	C-X-C Motif Chemokine Receptor 3
CXCR4	C-X-C Motif Chemokine Receptor 4
CXCR3/4	C-X-C Motif Chemokine Receptor 3/4
DNA	Deoxyribonucleic Acid
ERCP	Endoscopic Retrograde Cholangiopancreatography
FDR	False Discovery Rate
FOLFIRINOX	Folinic acid, Fluorouracil, Irinotecan, Oxaliplatin
h	Hour(s)
IFN	Interferon
IL	Interleukin
IL-1RA	Interleukin-1 Receptor Antagonist
IL-31RA	Interleukin-31 Receptor A
IMSS	Instituto Mexicano del Seguro Social
IQR	Interquartile Range
ISSSTE	Instituto de Seguridad y Servicios Sociales de los Trabajadores del Estado
KRAS	Kirsten Rat Sarcoma viral oncogene homolog
MAPK	Mitogen-Activated Protein Kinase
min	Minute(s)
mL	Milliliter(s)
MMP-9	Matrix Metallopeptidase 9
MYC	MYC Proto-Oncogene
NF-κB	Nuclear Factor Kappa B
OSMRβ	Oncostatin M Receptor Beta
PDAC	Pancreatic Ductal Adenocarcinoma
pg/mL	Picograms per milliliter
RNA	Ribonucleic Acid
ROC	Receiver Operating Characteristic
SD	Standard Deviation
STING	Stimulator of Interferon Genes
Th	T-helper cell
TNF	Tumor Necrosis Factor
TNFR1	Tumor Necrosis Factor Receptor 1
Tregs	Regulatory T cells
VEGF	Vascular Endothelial Growth Factor
WBC	White Blood Cell Count
