# Supplementary material for: Serum Cytokine Profiling in Pancreatic Ductal Adenocarcinoma: A Pilot Study of Candidate Discriminators, Sex Differences and Altered Co-Regulation
Source: Biomolecules. 2026 Oct 14;16(10):1492. doi: 10.3390/biom16101492 (PMC13702192; doi:10.3390/biom16101492)
Supplement: Supplementary file 1 [file biomolecules-16-01492-s001.zip › Supp_Fig2_AllCytokines_Female.pdf]

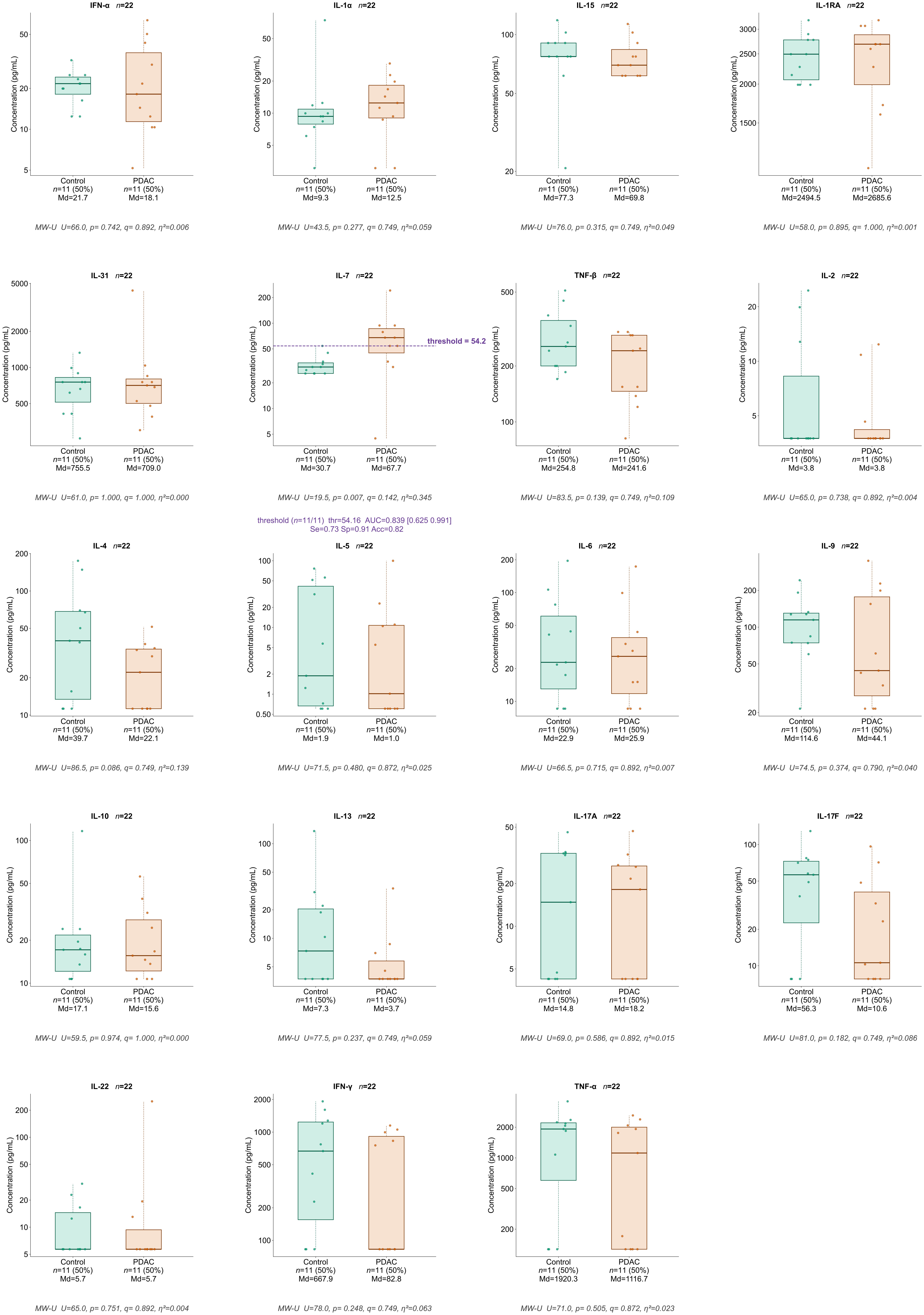
IFN-α n=22
Concentration (pg/mL)
Control n=11 (50%) Md=21.7
PDAC n=11 (50%) Md=18.1
MW-U U=66.0, p= 0.742, q= 0.892, η²=0.006
IL-1α n=22
Control n=11 (50%) Md=9.3
PDAC n=11 (50%) Md=12.5
MW-U U=43.5, p= 0.277, q= 0.749, η²=0.059
IL-15 n=22
Control n=11 (50%) Md=77.3
PDAC n=11 (50%) Md=69.8
MW-U U=76.0, p= 0.315, q= 0.749, η²=0.049
IL-1RA n=22
Control n=11 (50%) Md=2494.5
PDAC n=11 (50%) Md=2685.6
MW-U U=58.0, p= 0.895, q= 1.000, η²=0.001
IL-31 n=22
Control n=11 (50%) Md=755.5
PDAC n=11 (50%) Md=709.0
MW-U U=61.0, p= 1.000, q= 1.000, η²=0.000
IL-7 n=22
threshold = 54.2
Control n=11 (50%) Md=30.7
PDAC n=11 (50%) Md=67.7
MW-U U=19.5, p= 0.007, q= 0.142, η²=0.345
threshold (n=11/11) thr=54.16 AUC=0.839 [0.625 0.991]
Se=0.73 Sp=0.91 Acc=0.82
TNF-β n=22
Control n=11 (50%) Md=254.8
PDAC n=11 (50%) Md=241.6
MW-U U=83.5, p= 0.139, q= 0.749, η²=0.109
IL-2 n=22
Control n=11 (50%) Md=3.8
PDAC n=11 (50%) Md=3.8
MW-U U=65.0, p= 0.738, q= 0.892, η²=0.004
IL-4 n=22
Control n=11 (50%) Md=39.7
PDAC n=11 (50%) Md=22.1
MW-U U=86.5, p= 0.086, q= 0.749, η²=0.139
IL-5 n=22
Control n=11 (50%) Md=1.9
PDAC n=11 (50%) Md=1.0
MW-U U=71.5, p= 0.480, q= 0.872, η²=0.025
IL-6 n=22
Control n=11 (50%) Md=22.9
PDAC n=11 (50%) Md=25.9
MW-U U=66.5, p= 0.715, q= 0.892, η²=0.007
IL-9 n=22
Control n=11 (50%) Md=114.6
PDAC n=11 (50%) Md=44.1
MW-U U=74.5, p= 0.374, q= 0.790, η²=0.040
IL-10 n=22
Control n=11 (50%) Md=17.1
PDAC n=11 (50%) Md=15.6
MW-U U=59.5, p= 0.974, q= 1.000, η²=0.000
IL-13 n=22
Control n=11 (50%) Md=7.3
PDAC n=11 (50%) Md=3.7
MW-U U=77.5, p= 0.237, q= 0.749, η²=0.059
IL-17A n=22
Control n=11 (50%) Md=14.8
PDAC n=11 (50%) Md=18.2
MW-U U=69.0, p= 0.586, q= 0.892, η²=0.015
IL-17F n=22
Control n=11 (50%) Md=56.3
PDAC n=11 (50%) Md=10.6
MW-U U=81.0, p= 0.182, q= 0.749, η²=0.086
IL-22 n=22
Control n=11 (50%) Md=5.7
PDAC n=11 (50%) Md=5.7
MW-U U=65.0, p= 0.751, q= 0.892, η²=0.004
IFN-γ n=22
Control n=11 (50%) Md=667.9
PDAC n=11 (50%) Md=82.8
MW-U U=78.0, p= 0.248, q= 0.749, η²=0.063
TNF-α n=22
Control n=11 (50%) Md=1920.3
PDAC n=11 (50%) Md=1116.7
MW-U U=71.0, p= 0.505, q= 0.872, η²=0.023
