# Supplementary material for: Serum Cytokine Profiling in Pancreatic Ductal Adenocarcinoma: A Pilot Study of Candidate Discriminators, Sex Differences and Altered Co-Regulation
Source: Biomolecules. 2026 Oct 14;16(10):1492. doi: 10.3390/biom16101492 (PMC13702192; doi:10.3390/biom16101492)
Supplement: Supplementary file 1 [file biomolecules-16-01492-s001.zip › Supp_Fig3_AllCytokines_Male.pdf]

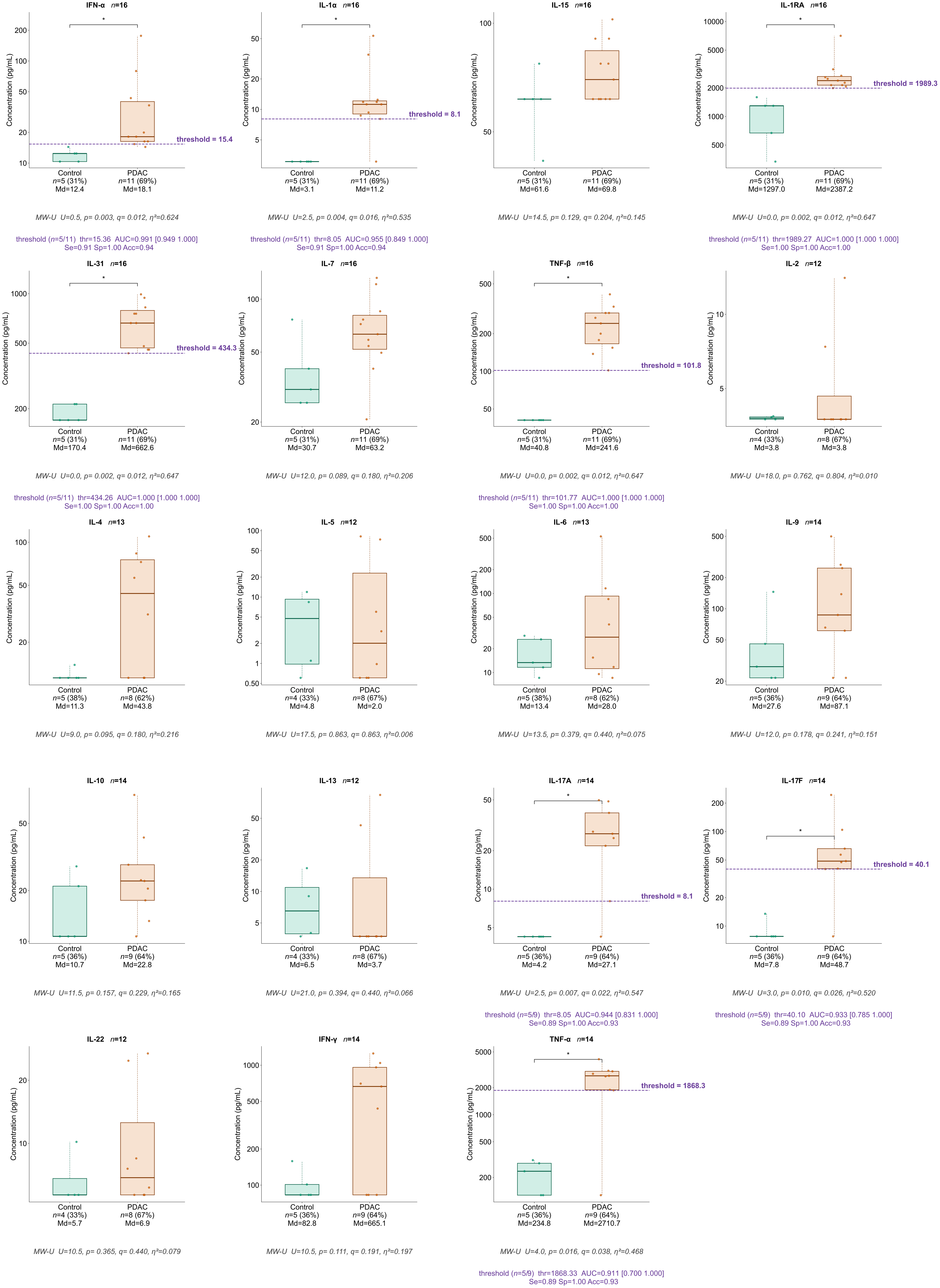

IFN-α n=16
*
threshold = 15.4
Control n=5 (31%) Md=12.4
PDAC n=11 (69%) Md=18.1
MW-U U=0.5, p= 0.003, q= 0.012, η²=0.624
threshold (n=5/11) thr=15.36 AUC=0.991 [0.949 1.000]
Se=0.91 Sp=1.00 Acc=0.94
IL-1α n=16
*
threshold = 8.1
Control n=5 (31%) Md=3.1
PDAC n=11 (69%) Md=11.2
MW-U U=2.5, p= 0.004, q= 0.016, η²=0.535
threshold (n=5/11) thr=8.05 AUC=0.955 [0.849 1.000]
Se=0.91 Sp=1.00 Acc=0.94
IL-15 n=16
Control n=5 (31%) Md=61.6
PDAC n=11 (69%) Md=69.8
MW-U U=14.5, p= 0.129, q= 0.204, η²=0.145
IL-1RA n=16
*
threshold = 1989.3
Control n=5 (31%) Md=1297.0
PDAC n=11 (69%) Md=2387.2
MW-U U=0.0, p= 0.002, q= 0.012, η²=0.647
threshold (n=5/11) thr=1989.27 AUC=1.000 [1.000 1.000]
Se=1.00 Sp=1.00 Acc=1.00
IL-31 n=16
*
threshold = 434.3
Control n=5 (31%) Md=170.4
PDAC n=11 (69%) Md=662.6
MW-U U=0.0, p= 0.002, q= 0.012, η²=0.647
threshold (n=5/11) thr=434.26 AUC=1.000 [1.000 1.000]
Se=1.00 Sp=1.00 Acc=1.00
IL-7 n=16
Control n=5 (31%) Md=30.7
PDAC n=11 (69%) Md=63.2
MW-U U=12.0, p= 0.089, q= 0.180, η²=0.206
TNF-β n=16
*
threshold = 101.8
Control n=5 (31%) Md=40.8
PDAC n=11 (69%) Md=241.6
MW-U U=0.0, p= 0.002, q= 0.012, η²=0.647
threshold (n=5/11) thr=101.77 AUC=1.000 [1.000 1.000]
Se=1.00 Sp=1.00 Acc=1.00
IL-2 n=12
Control n=4 (33%) Md=3.8
PDAC n=8 (67%) Md=3.8
MW-U U=18.0, p= 0.762, q= 0.804, η²=0.010
IL-4 n=13
Control n=5 (38%) Md=11.3
PDAC n=8 (62%) Md=43.8
MW-U U=9.0, p= 0.095, q= 0.180, η²=0.216
IL-5 n=12
Control n=4 (33%) Md=4.8
PDAC n=8 (67%) Md=2.0
MW-U U=17.5, p= 0.863, q= 0.863, η²=0.006
IL-6 n=13
Control n=5 (38%) Md=13.4
PDAC n=8 (62%) Md=28.0
MW-U U=13.5, p= 0.379, q= 0.440, η²=0.075
IL-9 n=14
Control n=5 (36%) Md=27.6
PDAC n=9 (64%) Md=87.1
MW-U U=12.0, p= 0.178, q= 0.241, η²=0.151
IL-10 n=14
Control n=5 (36%) Md=10.7
PDAC n=9 (64%) Md=22.8
MW-U U=11.5, p= 0.157, q= 0.229, η²=0.165
IL-13 n=12
Control n=4 (33%) Md=6.5
PDAC n=8 (67%) Md=3.7
MW-U U=21.0, p= 0.394, q= 0.440, η²=0.066
IL-17A n=14
*
threshold = 8.1
Control n=5 (36%) Md=4.2
PDAC n=9 (64%) Md=27.1
MW-U U=2.5, p= 0.007, q= 0.022, η²=0.547
threshold (n=5/9) thr=8.05 AUC=0.944 [0.831 1.000]
Se=0.89 Sp=1.00 Acc=0.93
IL-17F n=14
*
threshold = 40.1
Control n=5 (36%) Md=7.8
PDAC n=9 (64%) Md=48.7
MW-U U=3.0, p= 0.010, q= 0.026, η²=0.520
threshold (n=5/9) thr=40.10 AUC=0.933 [0.785 1.000]
Se=0.89 Sp=1.00 Acc=0.93
IL-22 n=12
Control n=4 (33%) Md=5.7
PDAC n=8 (67%) Md=6.9
MW-U U=10.5, p= 0.365, q= 0.440, η²=0.079
IFN-γ n=14
Control n=5 (36%) Md=82.8
PDAC n=9 (64%) Md=665.1
MW-U U=10.5, p= 0.111, q= 0.191, η²=0.197
TNF-α n=14
*
threshold = 1868.3
Control n=5 (36%) Md=234.8
PDAC n=9 (64%) Md=2710.7
MW-U U=4.0, p= 0.016, q= 0.038, η²=0.468
threshold (n=5/9) thr=1868.33 AUC=0.911 [0.700 1.000]
Se=0.89 Sp=1.00 Acc=0.93
Concentration (pg/mL)
