# Supplementary material for: Serum Cytokine Profiling in Pancreatic Ductal Adenocarcinoma: A Pilot Study of Candidate Discriminators, Sex Differences and Altered Co-Regulation
Source: Biomolecules. 2026 Oct 14;16(10):1492. doi: 10.3390/biom16101492 (PMC13702192; doi:10.3390/biom16101492)
Supplement: Supplementary file 1 [file biomolecules-16-01492-s001.zip › Supp_Fig1_AllCytokines_AllPatients.pdf]

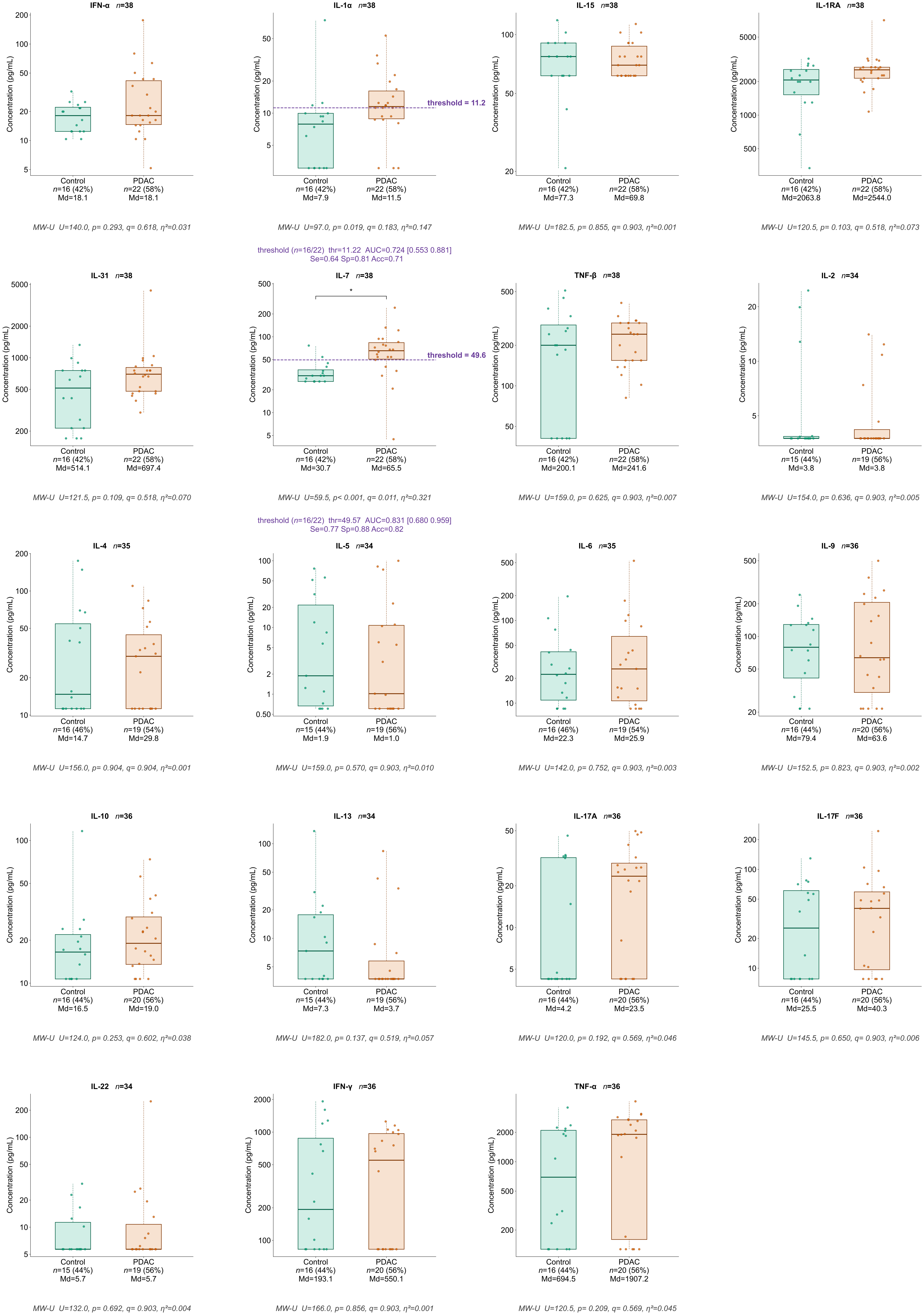
IFN-α n=38
Concentration (pg/mL)
Control n=16 (42%) Md=18.1
PDAC n=22 (58%) Md=18.1
MW-U U=140.0, p= 0.293, q= 0.618, η²=0.031
IL-1α n=38
threshold = 11.2
Control n=16 (42%) Md=7.9
PDAC n=22 (58%) Md=11.5
MW-U U=97.0, p= 0.019, q= 0.183, η²=0.147
threshold (n=16/22) thr=11.22 AUC=0.724 [0.553 0.881]
Se=0.64 Sp=0.81 Acc=0.71
IL-15 n=38
Control n=16 (42%) Md=77.3
PDAC n=22 (58%) Md=69.8
MW-U U=182.5, p= 0.855, q= 0.903, η²=0.001
IL-1RA n=38
Control n=16 (42%) Md=2063.8
PDAC n=22 (58%) Md=2544.0
MW-U U=120.5, p= 0.103, q= 0.518, η²=0.073
IL-31 n=38
Control n=16 (42%) Md=514.1
PDAC n=22 (58%) Md=697.4
MW-U U=121.5, p= 0.109, q= 0.518, η²=0.070
IL-7 n=38
*
threshold = 49.6
Control n=16 (42%) Md=30.7
PDAC n=22 (58%) Md=65.5
MW-U U=59.5, p< 0.001, q= 0.011, η²=0.321
threshold (n=16/22) thr=49.57 AUC=0.831 [0.680 0.959]
Se=0.77 Sp=0.88 Acc=0.82
TNF-β n=38
Control n=16 (42%) Md=200.1
PDAC n=22 (58%) Md=241.6
MW-U U=159.0, p= 0.625, q= 0.903, η²=0.007
IL-2 n=34
Control n=15 (44%) Md=3.8
PDAC n=19 (56%) Md=3.8
MW-U U=154.0, p= 0.636, q= 0.903, η²=0.005
IL-4 n=35
Control n=16 (46%) Md=14.7
PDAC n=19 (54%) Md=29.8
MW-U U=156.0, p= 0.904, q= 0.904, η²=0.001
IL-5 n=34
Control n=15 (44%) Md=1.9
PDAC n=19 (56%) Md=1.0
MW-U U=159.0, p= 0.570, q= 0.903, η²=0.010
IL-6 n=35
Control n=16 (46%) Md=22.3
PDAC n=19 (54%) Md=25.9
MW-U U=142.0, p= 0.752, q= 0.903, η²=0.003
IL-9 n=36
Control n=16 (44%) Md=79.4
PDAC n=20 (56%) Md=63.6
MW-U U=152.5, p= 0.823, q= 0.903, η²=0.002
IL-10 n=36
Control n=16 (44%) Md=16.5
PDAC n=20 (56%) Md=19.0
MW-U U=124.0, p= 0.253, q= 0.602, η²=0.038
IL-13 n=34
Control n=15 (44%) Md=7.3
PDAC n=19 (56%) Md=3.7
MW-U U=182.0, p= 0.137, q= 0.519, η²=0.057
IL-17A n=36
Control n=16 (44%) Md=4.2
PDAC n=20 (56%) Md=23.5
MW-U U=120.0, p= 0.192, q= 0.569, η²=0.046
IL-17F n=36
Control n=16 (44%) Md=25.5
PDAC n=20 (56%) Md=40.3
MW-U U=145.5, p= 0.650, q= 0.903, η²=0.006
IL-22 n=34
Control n=15 (44%) Md=5.7
PDAC n=19 (56%) Md=5.7
MW-U U=132.0, p= 0.692, q= 0.903, η²=0.004
IFN-γ n=36
Control n=16 (44%) Md=193.1
PDAC n=20 (56%) Md=550.1
MW-U U=166.0, p= 0.856, q= 0.903, η²=0.001
TNF-α n=36
Control n=16 (44%) Md=694.5
PDAC n=20 (56%) Md=1907.2
MW-U U=120.5, p= 0.209, q= 0.569, η²=0.045
